# A Tractable and Efficient One-Pot Synthesis of 5'-Azido-5'-deoxyribonucleosides

**DOI:** 10.3390/molecules19022434

**Published:** 2014-02-21

**Authors:** Theodore V. Peterson, Tobin U. B. Streamland, Ahmed M. Awad

**Affiliations:** Chemistry Program, California State University Channel Islands, One University Drive, Camarillo, CA 93012, USA; E-Mails: peterson.tv@gmail.com (T.V.P.); tobin.streamland@gmail.com (T.U.B.S.)

**Keywords:** modified nucleosides, 5'-azido nucleosides, Appel reaction, Mitsunobu reaction, antisense oligonucleotides, ribonucleic guanidine (RNG)

## Abstract

Synthetic routes to 5'-azidoribonucleosides are reported for adenosine, cytidine, guanosine, and uridine, resulting in a widely applicable one-pot methodology for the synthesis of these and related compounds. The target compounds are appropriate as precursors in a variety of purposive syntheses, as the synthetic and therapeutic relevance of azido- and amino-modified nucleosides is expansive. Furthermore, in the conversion of alcohols to azides, these methods offer a tractable alternative to the Mitsunobu and other more difficult reactions.

## 1. Introduction

The synthesis and chemical modification of nucleosides is a major research topic in medicinal and bioorganic chemistry. The vast utilization of nucleosides in most aspects of cellular function makes them a tantalizing target for a variety of therapies with potentially wide ranging physiological and pharmacological effects [1,2,3,4]. The molecular binding of nucleotide oligomers to highly specific targets as well as more heterogeneous binding has been shown to rival the affinity exhibited by antibodies both *in vitro* and *in vivo* [1,5,6,7]. This strong binding affinity and the generally low level of immunogenicity observed with nucleotide therapeutics uniquely position them to move quickly from the research bench to clinical applications [3,8]. The use of modified nucleosides as enzyme inhibitors, diagnostic reporters, and as anticancer or antiviral drugs is well documented in the literature [3,4,9,10,11], illustrating the extensive utility of these molecules. As the discovery of pharmacological targets increases the demand for synthetic analogs of nucleic acid monomers and oligomers, efficient reactions with wide-ranging biological applicability will be emphasized.

The azide functional group is among the most useful in organic synthesis, owing to its superlative reactivity. A great body of work has been done on azidonucleotides in particular, and although their bioactivity has been found to be negligible in many instances, they remain an important intermediate in a variety of purposive syntheses [12]. Nucleosides substituted with azide moieties are uniquely suited to combinable applications in click chemistry [13,14,15,16,17] and bio-conjugation (e.g., Staudinger ligation [18,19,20,21]) due to their reactivity and stability under physiological conditions. Furthermore, in conjugation reactions, azides confer a measure of kinetic control that overcomes the inability to control carbonyl chemistry with the efficiency nature exhibits [22]. In addition to these combinatorial applications and their fundamental use in the synthesis of heterocyclic compounds, azides are perhaps the most convenient source of naturally and pharmacologically pervasive amines [23,24,25].

Most contemporary alcohol to azide syntheses rely on two-step protocols proceeding through a halogenated intermediate, Mitsunobu-type displacement reactions, or other tenuous methodologies employing toxic, complex and/or expensive reagents [26,27,28]. However, the Appel reaction remains a simple reliable way to convert alcohols into to alkyl halides, and further substitution of this product by an azide salt produces azides in suitable yields. It has previously been reported that these two reactions can be combined affording azides from primary and secondary alcohols in one convenient synthesis [28,29]. Because Staudinger reduction of the resulting azide to a primary amine is a pervasive synthetic fate of azides, further reports have surfaced of a one-pot synthesis of amines directly from both primary and secondary alcohols, utilizing an Appel-Staudinger combinational approach [30]. The utility of a direct alcohol to amine conversion has spurned other approaches with equivalent goals [31,32]. However except for a few examples [28,29,33,34,35], research focusing on nucleic acids has largely ignored these routes to azides and amines, with syntheses proceeding through a Mitsunobu-type reaction seeming to be the preferred route. In reports utilizing azide addition as described by Hata, Yamamoto, and Sekine [36], application of the method resulted in low or unreported yields of modified nucleosides, with application to guanosine conspicuously absent. In contrast, we report in the present study a highly efficient one-pot synthetic approach to produce both purine and pyrimidine 5'-azido ribonucleosides. Our one-pot synthetic route relies on readily available compounds and mild reaction conditions, allowing synthesis of azide-substituted nucleosides in suitable yields for follow-on syntheses without the reliance on Mitsunobu and its previously mentioned issues.

## 2. Results and Discussion

**Figure 1:**
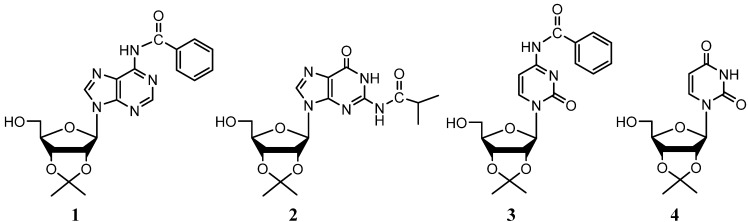
Ribonucleosides analogs used for the one-pot synthesis of 5'-azido nucleosides.

Protected ribonucleosides utilized as starting material were synthesized in two steps, except for uridine. Adenosine, cytidine and guanosine first require protection of the *exo*-cyclic amine moieties found in their respective nitrogenous bases prior to selective substitution of the 5'-hydroxyl group. Subsequent protection of the 2' and 3' hydroxyl groups of each nucleoside to produce starting material compounds **1**–**4** (Figure 1) was performed in anhydrous acetone with 2,2-dimethoxypropane in the presence of an acid catalyst. The reaction mixture was refluxed until complete disappearance of the starting material, as monitored by TLC. While analogs of adenosine, guanosine and cytidine required significant times at reflux temperature, the yield of **4** was higher when reacted at room temperature. Nearly pure products of **2** and **3** were obtained after a bicarbonate wash and evaporation of the reaction mixture, however in the case of **1** and **4**, flash column chromatography was required for final purification.

**Scheme 1:**
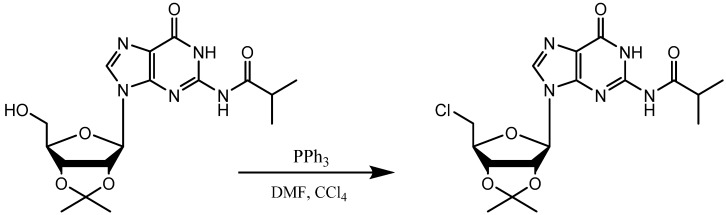
Appel reaction product: 5'-chloro-2',3'-O-isopropylideneguanosine.

Initially we were interested in the direct synthesis of 5'-amino substituted nucleosides via a one-pot combined Appel-Staudinger mechanism, but modeling our approach on work done with other alcohols [30,32] did not produce the desired nucleoside product in easily recoverable yields. Elemental analysis by ESI mass spectrometry indicated that our initial approach produced a 5'-chloro nucleoside, the expected Appel product (Scheme 1).

**Scheme 2:**
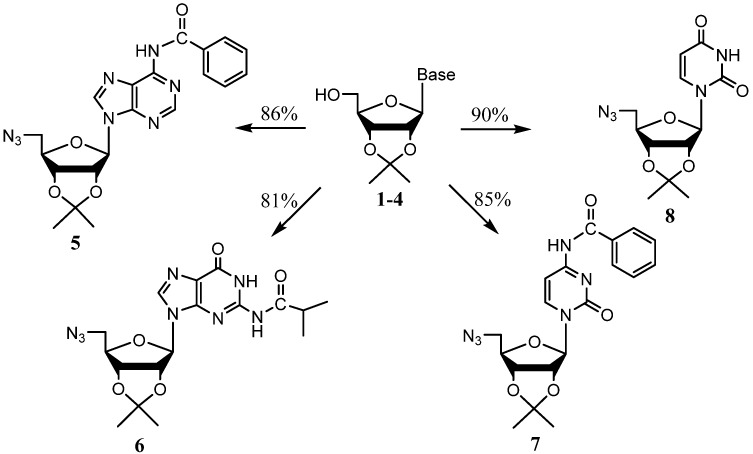
General synthesis of 5'-azido ribonucleosides.

Heating the protected nucleosides with CBr_4_ as a halide source in the presence of triphenylphosphine and an excess of NaN_3_ favored the formation of 5'-azidonucleosides in high yields (Scheme 2).

**Scheme 3:**
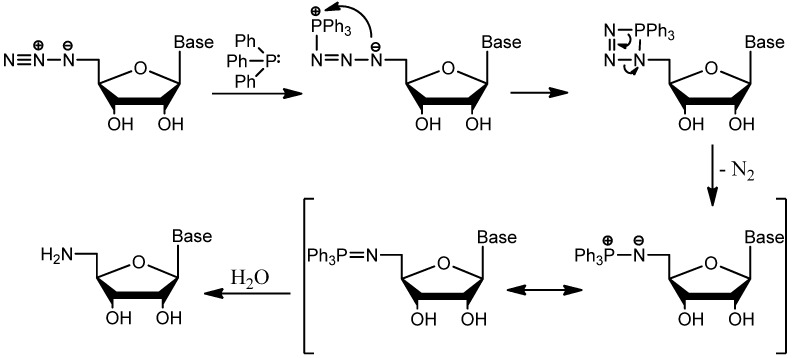
Reduction of azides to amines by Staudinger reaction.

Staudinger reduction of azides requires excess triphenylphosphine to form the iminophosphorane intermediate (Scheme 3), which is then hydrolyzed to form the corresponding amino derivative. However, using excess PPh_3_ did not produce the 5'-aminonucleoside. As noted by Hata *et al.* and observed in our syntheses, excess PPh_3_ or alkyl halide concentration results in a mixture of products and a more difficult extraction [29]. Reducing the molar contribution of PPh_3_ in this one-pot synthesis ensures that: (i) after creation of the initial Appel product and subsequent substitution by azide, no PPh_3_ remains to form the said intermediate; and (ii) product recovery is significantly improved due to a reduction in the amount of phosphonium oxide by-product formed. Applying this reduced-reagent one-pot technique to the protected starting material generated from adenosine, guanosine, cytidine, and uridine afforded 5'-azido-5'-deoxyribonucleosides in high yields for application as intermediates and precursors in a variety of nucleoside syntheses.

*Reagents and conditions*: Protected nucleosides in anhydrous DMF, PPh_3_, CBr_4_, NaN_3_, heat at 90 °C.

**Figure 2:**
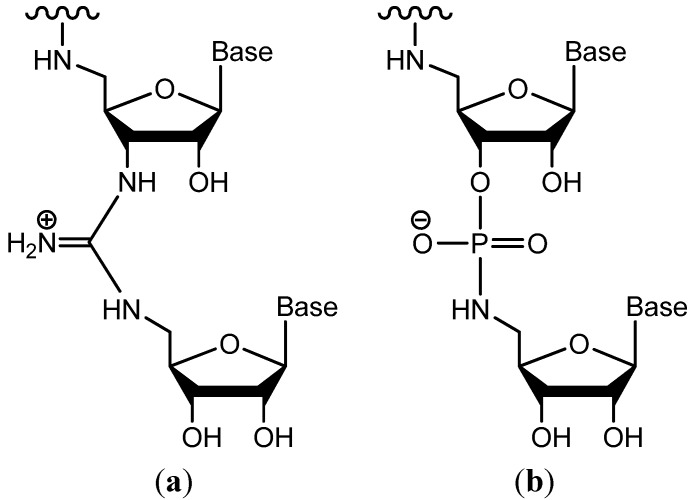
(**a**) Ribonucleic guanidine (RNG); (**b**) P3'→N5' phophoramidate RNA.

Our interest in 5'-azido- and 5'-aminonucleoside analogs stems primarily from their application as precursors in the synthesis of positively charged guanidine-linked ribooligonucleotides (RNG in Figure 2a) and P3'→N5' phosphoramidate ribonucleotides (Figure 2b). We have previously reported synthetic routes to 3' and 5'-terminal monomers of cytidine and uridine required for these applications [37,38]. Notably, synthesis of the 3'-terminal monomer utilized the Mitsunobu reaction to facilitate the eventual amination of the 5' moiety. However, adaptation of this method in related syntheses for guanosine proved largely ineffective. Of the four most common nucleobases found in RNA, guanine has been acknowledged to increase experimental problems. In our experience, this character is further exasperated in Mitsunobu-type reactions, in which there is substantial potential for reaction interference. To avoid this in the present study, we decided to explore alternatives and abandon the Mitsunobu approach for all nucleosides included here.

One pot reactions were instead prepared from **1**–**4** in the minimum amount of anhydrous DMF and then allowed to react with PPh_3_, CBr_4_, and NaN_3_ added in order, allowing the previous reactant to completely dissolve before proceeding to the next. The reaction mixtures were stirred for 10 min at room temperature then stirred overnight at 90 °C. The products were purified by column chromatography to afford 5'-azidonucleotides **5**–**8** in excellent yields (81%–90%). Protection of the 2' and 3' hydroxyl groups seems to be vindicated as our one-pot yields exceeded those reported by Hata *et al.* [29] for two of the three bases our syntheses shared (Table 1).

**Table 1:** 5'-Azidonucleoside synthesis from base-protected nucleosides of A, G, C and U: comparison of synthetic approaches.

Nucleoside	Synthetic Path	Overall yield (One-pot Yield)	Hata *et al.* yields (One step rxn.)
N^6^-Benzoyladenosine	*1→5*	74.0% (86%)	56%
N^2^-Isobutyrylguanosine	*2→6*	68.0% (81%)	N/A
N^4^-Benzoylcytidine	*3→7*	77.3% (85%)	45%
Uridine	*4→8*	79.2% (90%)	92%

Although guanidinium linkages are of primary focus within our lab, further modification may reveal synthetic nucleoside analogues with the ability to treat or aid immune system response, whether through anti-viral or therapeutic methods. Nucleosides were of primary focus only several decades ago, and with advances in methodology and technology, therapeutic nucleosides will again move into the spotlight as agents of vast clinical potential.

## 3. Experimental

### General Procedures

^1^H-NMR spectra were recorded on a 500 MHz Varian instrument, using CDCl_3_ or DMSO-d_6_ as solvents*.* Chemical shifts were reported in δ ppm and coupling constants (*J*) are given in Hz*.* Thin-layer chromatography (TLC) was carried out on Silica Gel 60 F-254 pre-coated plates (Selecto Scientific, Suwanee, GA, USA and EMD Millipore, Merck KGaA, Darmstadt, Germany) and visualization of the products was performed under UV light. Silica gel used for flash column chromatography was Scientific Silica Gel (particle size 32–63, Selecto Scientific and/or particle size 38–63, Whatman International, Maidstone, Kent, UK). ESI mass spectra were recorded on QSTAR Pulsar Quadrupole/time-of-flight mass spectrometer with Turbo Ion Spray Ionization Source (Applied Biosystems, Foster City, CA, USA). All reactions were performed using anhydrous solvents under N_2_ atmosphere. All flash column chromatography performed used 3:1 ethyl acetate to hexanes for product isolation.

*N6-Benzoyl-2',3'-O-isopropylideneadenosine* (**1**). To a stirred solution of *N*^6^-benzoyladenosine (5.60 g, 15 mmol) in anhydrous acetone (100 mL) was added 2,2-dimethoxypropane (16.6 mL, 135 mmol) and *p-*toluenesulfonic acid monohydrate (0.15 g, 0.75 mmol). The reaction mixture was refluxed for 18 h, then filtered to remove any unreacted starting material. The filtrate was concentrated under vacuum and the residue was dissolved in EtOAc, washed with 5% NaHCO_3_, brine, and dried over sodium sulfate anhydrous. The solvent was removed under reduced pressure and the solid product was washed with diethyl ether to remove any residual 2,2-dimethoxypropane then filtered. The product was further purified by silica gel column chromatography (3:1 EtOAc to hexanes) to afford pure compound **1** (5.31 g, 86%). HRMS (ESI) *m/z* calcd. for C_20_H_21_N_5_O_5_ (M+Na)^+^ 434.1440, found 434.1441. NMR (DMSO-*d*_6_): 11.38 br s, 1H (NH); 8.91 s, 1H (H-8); 8.81 s, 1H (H-2); 8.18-7.67 m, 5H (H-Bz); 6.40 d, 1H, *J* = 11.8 (H-1'); 5.56 q, 1H, *J* = 8.3 (H-2'); 5.28 br s, 1H (OH-5'); 5.13 t, 1H, *J* = 5.9 (H-3'); 4.18 q, 1H, *J* = 6.3 (H-4'); 3.69–3.67 m, 2H (H-5'); 1.63 s, 3H (CH_3_); 1.25 s, 3H (CH_3_). 

*N2-Isobutyryl-2',3'-O-isopropylideneguanosine* (**2**). To a stirred solution of *N*^2^-isobutyrylguanosine (5.30 g, 15 mmol) in anhydrous acetone (100 mL) was added 2,2-dimethoxypropane (16.6 mL, 135 mmol) and *p-*toluenesulfonic acid monohydrate (0.15 g, 0.75 mmol). The reaction mixture was refluxed for 18 h. The solvent was removed under reduced pressure, and the residue was dissolved in EtOAc, washed with 5% NaHCO_3_, brine, and dried over sodium sulfate anhydrous. The solvent was removed under vacuum, and the residue was stirred for five minutes in ether then filtered to afford pure compound **2** (4.96 g, 84%). ^1^H-NMR (DMSO-*d*_6_): 12.10 s, 1H (NH); 11.54 s, 1H (NH); 7.83 s, 1H (H-8); 5.84 d, 1H, *J* = 2.8, (H-1'); 5.11 m, 1H (H-2'); 5.04 m, 1H (H-3'); 4.44 q, 1H, *J* = 3.9 (H-4'); 3.99–3.78 m, 2H, (H-5'); 2.78-2.76 m, 1H (CH); 1.62 s, 3H (CH_3_); 1.37 s, 3H (CH_3_); 1.28 d, 6H, *J* = 6.8 (C(CH_3_)_2_).

*N4-Benzoyl-2',3'-O-isopropylidenecytidine* (**3**). To a stirred suspension of *N*^4^-benzoylcytidine (5.20 g, 15 mmol) in anhydrous acetone (100 mL), *p*-toluenesulfonic acid monohydrate (0.15 g, 0.75 mmol) and 2-2-dimethyoxypropane (13.85 mL, 112.5 mmol) were added and the reaction mixture was refluxed overnight under N_2_ atmosphere. To remove the unreacted starting material, the mixture was filtered through Celite, which was then washed with acetone and methylene chloride. The filtrate was concentrated under vacuum and the residue was dissolved in EtOAc, washed with 5% NaHCO_3_, brine, and dried over sodium sulfate anhydrous. The solvent was removed under reduced pressure, and the residue was stirred for five minutes in ether then filtered to afford pure compound **3** (5.29 g, 91%). ^1^H- NMR (DMSO-*d*_6_): 11.22 br s, 1H (NH); 8.29 d, 1H, *J* = 7.8 (H-6); 8.00-7.51 m, 5H (BzH); 7.32 d, 1H, *J* = 7.2 (H-5); 5.85 d, 1H, *J* = 2.0 (H-1'); 5.12 t, 1H, *J* = 5.4 (OH-5'); 4.90 dd, 1H, *J* = 4.0, 1.9 (H-2'); 4.76 q, 1H, *J* = 3.4 (H-3'); 4.21 q, 1H, *J* = 3.5 (H-4'); 3.64-3.55 m, 2H (H-5'); 1.48 s, 3H (CH_3_); 1.28 s, 3H (CH_3_).

*2',3'-O-Isopropylideneuridine* (**4**). To a stirred solution of uridine (3.65 g, 15 mmol) in anhydrous acetone (100 mL) was added 2,2-dimethoxypropane (10.05 mL, 82 mmol) and *p-*toluenesulfonic acid monohydrate (0.15 g, 0.75 mmol). The reaction mixture was stirred overnight at room temperature. The solvent was removed under reduced pressure, and the residue was dissolved in EtOAc, washed with 5% NaHCO_3_, brine, and dried over sodium sulfate anhydrous. The solvent was removed under vacuum, and the crude was purified by silica gel column chromatography (3:1 ratio of EtOAc to hexanes) to afford compound **4** (3.75 g, 88%). ^1^H-NMR (DMSO-*d*_6_): 11.36 s 1H, (NH); 7.78 d, 1H, *J* = 8.3 (H-6); 5.81 d, 1H, *J* = 2.5 (H-1'); 5.62 d, 1H, *J* = 7.9 (H-5); 5.08 t, 1H, *J* = 5.4 (OH-5'); 4.88 q, 1H, *J* = 2.4 (H-2'); 4.73 q, 1H, *J* = 3.9 (H-3'); 4.06 q, 1H, *J* = 4.4 (H-4'); 3.57–3.52 m, 2H (H-5'); 1.46 s, 3H (CH_3_); 1.27 s, 3H (CH_3_).

*5'-Azido-N6-benzoyl-2',3'-O-isopropylidene-5'-deoxyadenosine* (**5**). To a stirred solution of compound **1** (4.00 g, 10 mmol) in anhydrous DMF (30 mL) was added PPh_3_ (3.95 g, 15 mmol) and CBr_4_ (6.00 g, 18 mmol). The mixture was stirred under N_2_ at room temperature for 5 min, then excess NaN_3_ (2.95 g, 45 mmol) was added. The reaction mixture was heated under N_2_ at 90 °C for 24 h. The reaction was quenched by addition of H_2_O (10 mL). After stirring for 5 min, the mixture was diluted with EtOAc (20 mL), washed with 5% NaHCO_3_, brine, and the organic layer was collected and dried over sodium sulfate anhydrous. The solvent was removed under reduced pressure and the crude product was purified by silica gel column chromatography (3:1 ratio of EtOAc to hexanes) to afford white solid of compound **5** (3.75 g, 86%). HRMS (ESI) *m/z* calcd. for C_20_H_20_N_8_O_4_ (M+Na)^+^ 459.1505, found 459.1490. ^1^H-NMR (CDCl_3_): 10.75 br s, 1H (NH); 8.99 s, 1H (H-8); 8.84 s, 1H (H-2); 8.16–7.26 m, 5H (H-Bz); 6.29 d, 1H, *J* = 11.8 (H-1'); 5.46 m, 1H (H-2'); 5.07 m, 1H (H-3'); 4.41 br s, 1H (OH-5'); 4.13 q, 1H, *J* = 6.9 (H-4'); 3.60 m, 2H (H-5'); 1.60 s, 3H (CH_3_); 1.25 s, 3H (CH_3_).

*5'-Azido-N2-isobutyryl-2',3'-O-isopropylidene-5'-deoxyguanosine* (**6**). To a stirred solution of compound **2** (3.95g, 10 mmol) in anhydrous DMF (30 mL) was added PPh_3_ (3.95 g, 15 mmol) and CBr_4_ (6.00 g, 18 mmol). The mixture was stirred under N_2_ at room temperature for 5 min, then excess NaN_3_ (2.95 g, 45 mmol) was added. The reaction mixture was heated under N_2_ at 90 °C for 24 h. The reaction was quenched by addition of H_2_O (10 mL). After stirring for 5 min, the mixture was diluted with EtOAc (20 mL), washed with 5% NaHCO_3_, brine, and the organic layer was collected and dried over sodium sulfate anhydrous. The solvent was removed under reduced pressure and the crude product was purified by silica gel column chromatography (3:1 ratio of EtOAc to hexanes) to afford a pale yellow solid of compound **6** (3.39 g, 81%). HRMS (ESI) *m/z* calcd. for C_17_H_22_N_8_O_5_ (M+Na)^+^ 441.1611, found 441.1591. ^1^H-NMR (CDCl_3_): 12.02 s, 1H (NH); 7.83 s, 1H (H-8); 5.99 d, 1H, *J* = 2.9 (H-1'); 5.15 q, 1H, *J* = 2.9 (H-2'); 4.94 q, 1H, *J* = 3.9 (H-3'); 4.35 q, 1H, *J* = 4.4 (H-4'); 3.65–3.51 m, 2H, (H-5'); 2.62-2.58 m, 1H (CH); 1.62 s, 3H (CH_3_); 1.39 s, 3H (CH_3_); 1.29 d, 6H, *J* = 6.8 (C(CH_3_)_2_).

*5'-Azido-N4-benzoyl-2',3'-O-isopropylidene-5'-deoxycytidine* (**7**). To a stirred solution of compound **3** (3.90g, 10 mmol) in anhydrous DMF (30 mL) was added PPh_3_ (3.15 g, 12 mmol) and CBr_4_ (6.00 g, 18 mmol). The mixture was stirred under N_2_ at room temperature for 5 min, then excess NaN_3_ (2.95 g, 45 mmol) was added. The reaction mixture was heated under N_2_ at 90 °C for 24 h. The reaction was quenched by addition of H_2_O (10 mL). After stirring for 5 min, the mixture was diluted with EtOAc (20 mL), washed with 5% NaHCO_3_, brine, and the organic layer was collected and dried over sodium sulfate anhydrous. The solvent was removed under reduced pressure and the crude product was purified by silica gel column chromatography (3:1 ratio of EtOAc to hexanes) to afford compound **7** (3.51 g, 85%). HRMS (ESI) *m/z* calcd. for C_19_H_20_N_6_O_5_ (M+Na)^+^ 435.1393, found 435.1375. ^1^H-NMR (CDCl_3_): 7.91 d, 1H, *J* = 7.3 (H-6); 7.63-7.51 m, 5H (BzH); 7.32 d, 1H, *J* = 7.2 (H-5); 5.70 d, 1H, *J* = 1.6 (H-1'); 4.91 dd, 1H, *J* = 4.4, 3.9 (H-2'); 4.34 m, 1H (H-3'); 4.14 q, 1H, *J* = 6.8 (H-4'); 3.77–3.63 m, 2H (H-5'); 1.58 s, 3H (CH_3_); 1.36 s, 3H (CH_3_).

*5'-Azido-2',3'-O-isopropylidene-5'-deoxyuridine* (**8**). To a stirred solution of compound **4** (2.85 g, 10 mmol) in anhydrous DMF (30 mL) was added PPh_3_ (3.15 g, 12 mmol) and CBr_4_ (6.00 g, 18 mmol). The mixture was stirred under N_2_ at room temperature for 5 min, then excess NaN_3_ (2.95 g, 45 mmol) was added. The reaction mixture was heated under N_2_ at 90 °C for 24 h. The reaction was quenched by addition of H_2_O (10 mL). After stirring for 5 min, the mixture was diluted with EtOAc (20 mL), washed with 5% NaHCO_3_, brine, and the organic layer was collected and dried over sodium sulfate anhydrous. The solvent was removed under reduced pressure and the crude product was purified by silica gel column chromatography (3:1 ratio of EtOAc to hexanes) to afford a white solid of compound **8** (2.78 g, 90%). HRMS (ESI) *m/z* calcd. for C_12_H_15_N_5_O_5_ (M+Na)^+^ 332.0971, found 332.0972. ^1^H-NMR (CDCl_3_): 9.30 s 1H, (NH); 7.30 d, 1H, *J* = 7.9 (H-6); 5.91 d, 1H (H-1'); 5.78 d, 1H, *J* = 7.8 (H-5); 5.00 d, 1H, *J* = 6.4 (H-2'); 4.82 t, 1H, *J* = 3.9 (H-3'); 4.24 q, 1H, *J* = 4.9 (H-4'); 4.13–3.62 m, 2H (H-5'); 1.56 s, 3H (CH_3_); 1.35 s, 3H (CH_3_).

## 4. Conclusions

We have confirmed the synthesis of 5'-azido-5'-deoxyribonucleosides of the bases adenine, guanine, cytosine, and uracil by means of ^1^H-NMR and high resolution mass spectrometry. Owing to the unique chemical characteristics of the azide functional group, these molecules have the potential to be used in applications even more diverse than their contemporary use. In particular, these molecules are available for solid-phase synthesis of ribonucleic acids with guanadium linkages.
